# Emerging features of MAIT cells and other unconventional T cell populations in human viral disease and vaccination.

**DOI:** 10.1016/j.smim.2022.101661

**Published:** 2022-11

**Authors:** Carl-Philipp Hackstein, Paul Klenerman

**Affiliations:** a Peter Medawar Building for Pathogen Research, Nuffield Dept of Medicine, University of Oxford, Oxford OX1 3SY, UK; b Translational Gastroenterology Unit, Nuffield Dept of Medicine, John Radcliffe Hospital, Oxford OX3 9DU, UK

**Keywords:** MAIT, Unconventional T cell, SARS-COV2, HIV, Viral infection

## Abstract

MAIT cells are one representative of a group of related unconventional or pre-set T cells, and are particularly abundant in humans. While these unconventional T cell types, which also include populations of Vδ2 cells and iNKT cells, recognise quite distinct ligands, they share functional features including the ability to sense “danger” by integration of cytokine signals. Since such signals are common to many human pathologies, activation of MAIT cells in particular has been widely observed. In this review we will discuss recent trends in these data, for example the findings from patients with Covid-19 and responses to novel vaccines. Covid-19 is an example where MAIT cell activation has been correlated with disease severity by several groups, and the pathways leading to activation are being clarified, but the overall role of the cells in vivo requires further exploration. Given the potential wide functional responsiveness of these cells, which ranges from tissue repair to cytotoxicity, and likely impacts on the activity of many other cell populations, defining the role of these cells - not only as sensitive biomarkers but also as mediators - across human disease remains an important task.

## 1 Unconventional T cells are diverse in phenotype and but share key functions

Non- MHC-restricted T lymphocytes are commonly referred to as unconventional T cells, an umbrella term covering a broad range of different cell populations including γδ T cells, CD1d-restriced natural killer T cells (NKT), MR1-restricted mucosal-associated invariant T cells (MAIT) and additional less well-studied T cells subsets responding to antigens presented by other CD1- and MHC-Ib molecules [1].

The role and functions of the different subsets of unconventional T cells have been extensively studied in a large variety of different pre-clinical models and clinical settings. However, due to the diverse nature and their broad partially overlapping and context-dependent functionality, the exact role(s) of unconventional T cells in several important processes are not fully understood.

The composition of the unconventional T cell compartment varies notably between species [2], [3]. In humans, MAIT cells represent the major unconventional population, followed by Vδ2-cells with MAIT-like characteristics, while other subsets like iNKT cells or CD1b-restricted GEM T cells are considerably rarer [4], [5], [6], [7]. In general, unconventional T cells have been implicated in a diverse set of clinically relevant settings ranging from tissue repair and the maintenance of tissue homeostasis, through anti-microbial immunity to autoimmune diseases and cancer. The multifaceted and sometimes conflicting roles of different unconventional T cells in cancer and different promising approaches of utilizing them in cancer therapy have been reviewed in detail recently [8], [9], [10]. Further, other parts of this review series are discussing specific roles of unconventional T cells like tissue repair functions and the role of unconventional T cell in anti-bacterial immunity. Hence, in this review, we will address what is known about unconventional T cells and particularly MAIT cells in the context of clinically relevant viral infections, focusing on the most recent data.

## 2 MAIT and other unconventional T cells can serve as biomarkers of inflammation

A defining feature of unconventional T cells is the high expression of a diverse range of receptor molecules, including NK cell, chemokine and cytokine receptors [11], [12]. Expression of these molecules is crucial for the innate-like properties of unconventional T cells as they allow them to respond independently of their TCRs.

The responsiveness of human MAIT cells to IL-12, IL-15, IL-18, IL-23 and type I interferons via the respective receptors is a prime example of this innate-like behaviour and constitutes the basis for MAIT cell activation in a variety of clinical settings associated with high expression of these cytokines (reviewed in [12]). Hence, one could consider MAIT cell activation as a biomarker to measure inflammation in settings like sepsis, inflammatory diseases and viral infections.

Viruses are not known to express any antigens activating MAIT or iNKT cells in a TCR-dependent manner. However, elevated levels of various cytokines including IL-12, IL-15, IL-18 or type I interferon are commonly seen in the context of viral infection. In most of the settings studied, signals from multiple cytokines must be integrated in order to provide effective MAIT cell activation (e.g. cytokine release), although some partial activation (e.g. CD69 upregulation) can be seen with individual triggers. Cytokine-dependent activation of MAITs has been shown in a range of clinically relevant viral infections including Dengue fever (DENV), influenza (IAV), HCV [13], HIV [14], endemic Hantavirus [15] and recently Sars-Cov2 (see below). Interestingly, the importance of different cytokines for TCR-independent MAIT cell activation seems partially to depend on the viral infection, e.g. IL-12 was required for maximal MAIT activation in response to DENV but not IAV or HCV, while blocking IL-15 only showed an impact on the response in a HCV-context. In contrast, virally induced IL-18 was shown to play a role in every of these three settings [13] and in an *in vitro* HIV model [16]. The response in the context of Hantavirus infection was dependent on type I Interferon [15], a feature also seen in influenza in humans and mice [13], [17].

## 3 Human viral infections activate and deplete unconventional T cells during chronic viral infection

Chronic HIV: The impact of HIV infection, acutely and during anti-retroviral therapy (ART), on unconventional T cells has been analysed in a large body of clinical studies, concluding that the infections perturbs the frequencies of circulating MAIT and iNKT cells [18] as well as γδ T cells [19]. MAIT cells frequencies were consistently reported to be reduced during chronic HIV infection [14], [20], [21] in blood and do not recover upon undergoing ART therapy [16], [20]. Interestingly, even in the chronic phase the residual MAIT cells seem to be highly activated as indicated by the upregulation of PD-1, TIM3 and GzmB [16]. HIV-induced activation of MAIT cells was dependent on IL12 and IL-18, and CCL3,4 and 5- dependent anti-HIV effects could be shown in in vitro models. These latter chemokines possess potent effects on HIV spread suggesting that MAIT cells might contribute to antiviral immunity in vivo [16].

Like MAIT cells, iNKT were reported to be diminished in different tissues in HIV-infected individuals [18], [22], [23], display a more activated phenotype [24] and increased expression of co-inhibitory surface receptors [25]. In contrast to MAIT cells, a major subset of iNKTs expresses the CD4 co-receptor and hence is susceptible to be directly infected with HIV. Consistently, it was reported that particularly CD4 + iNKTs are depleted during HIV infection and that depletion already takes place during the acute phase of the disease [26]. Within the γδ T cell population, HIV infection is associated with a reduction of Vδ2 and an increase in Vδ1 T cells [27], [28], [29], [30]. Again, as observed in the MAIT and iNKT populations, human Vδ2 from HIV patients display a more activated phenotype than their counterparts from health controls [29], [31] and the HIV-induced changes seem to be long-lasting and do not normalise upon successful ART therapy [32].

Hepatitis C Virus: Similar observations were made in the context of HCV infection and HIV/HCV co-infection, featuring a loss of MAIT cells from the circulation, a highly activated, dysfunctional phenotype and do lack of recovery upon therapy [20], [33], [34], [35]. Hepatic MAIT cell frequencies are reduced in HIV patients and are inversely correlated with hepatic inflammation and fibrosis [36], however currently no studies addressed whether the former is due to an actual depletion of the cells or the results of a dilution of the MAIT cell population by infiltrating conventional T cells. As in vitro experiments suggest that MAIT cells have an IFNγ-dependent antiviral effect [13], future studies are needed to fully elucidate the roles of MAIT cells in HCV infection.

Hepatitis B and D viruses: Most [37], [38], [39] but not all [40] reports conclude that peripheral MAIT cells also decrease in chronic HBV infection. Similar to HIV and HCV, HBV infection is consistently associated with increased expression of markers like HLA-DR, CD38, PD-1 and other activation and co-inhibitory surface markers as well as reduced functional capacity. Co-infection of HBV with HDV seems to have a more pronounced impact on MAIT cells in both, the periphery and the liver itself, compared to HBV-monoinfection [41].

Human T-cell leukaemia virus 1: HTLV-1 infection also induces activation and dysfunction in MAIT cells and, regardless of whether is it symptomatic or not, is associated with reduced MAIT frequencies in the peripheral blood [42]. Notably, MAIT cells were less likely to be infected with HTLV-1 compared to CD4 T cells suggesting that the deletion is not caused directly by the virus.

Overall, chronic viral infections have deleterious effects on unconventional T cells in general and MAIT cells in particular resulting in strong activation, exhaustion and deletion of these cells. Interestingly these effects are long-lasting in the blood, even under therapy, limiting the usefulness of unconventional T cells as biomarkers in chronic settings. However, as illustrated by the inverse correlation of MAIT cell frequencies and disease state in the liver during HCV infection, the analysis of tissue-resident MAIT and other unconventional T cells could have important implications for prognosis of clinical relevant disease.

Studies in mouse models of fibrosis have recently indicated that MAIT cells play a role in driving this via cross-talk with hepatic stellate cells [43], [44]. On the other hand in a normal liver MAITs are exposed continuously to TCR-triggering via transport of the ligand in the portal vein [45] potentially allowing for ongoing homeostatic control via the “tissue repair” programme. How the depletion and cytokine mediated activation of MAIT cells is disrupted in chronic viral infections to promote fibrosis is not known, although one potential explanation is that loss/dysfunction of the cells limits the capacity for normal tissue homeostasis/repair in this organ [46]. Given the strong association of MAIT cells with the liver and the importance of liver fibrosis in chronic viral infection, this issue requires further clarification.

## 4 MAIT cell activation mirrors disease status during acute viral infections

### 4.1 Acute HIV

Interestingly, while MAIT cells are depleted during chronic HIV infection, they get highly activated and actually expand early during the acute phase of the disease [47]. Peaks of MAIT cell activation and expansion were slightly delayed compared to the viral load and activation correlated well with the amount of soluble CD14 in the plasma, potentially reflecting the extent of microbial translocation in patients.

### 4.2 Dengue and influenza

MAIT cells have been studied in the context of several acute viral infections including Dengue fever and influenza. Both infections lead to a transient activation of the MAIT cell population as indicated by upregulation of markers like CD38 and GzmB and perturbation of MAIT cell numbers [13], [48]. Importantly, these changes correlate with disease severity as a more pronounced phenotype was observed in patients suffering from Dengue haemorrhagic fever than from those with milder disease and in IAV patients who required intensive care compared to those who did not [13]. This is also corroborated by a retrospective study of patients hospitalized due to avian IAV H7N9 which found that peripheral MAIT cells numbers correlated with the likelihood of survival and a quick recovery [49], suggesting that MAIT cells could contribute to the anti-IAV immune response and might represent an important clinical parameter.

### 4.3 Hantavirus

Activation of MAIT cells was recently also reported in the context of infection with Puumala orthohantavirus (PUUV), the causative agent of haemorrhagic fever with renal syndrome (HFRS). Importantly, in the case of HFRS, MAIT cell activation was shown to correlate with disease severity [15], which would allow its use as a biomarker in the context of this disease.

### 4.4 Covid-19

MAIT cell activation during virus infection was a particularly striking finding during the SARS-CoV-2 pandemic. A number of papers have described overlapping – in some cases identical – findings regarding the features of activation first observed in influenza, dengue and HIV above [50], [51], [52], [53], [54], [55], [56], [57], [58], [59], [60], [61], [62]. The earliest paper to describe this came from analyses of severely ill patients and included both blood and studies of lung derived cells (BAL) [43]. A very striking activation of MAITs was seen in parallel to iNKTs, accompanied as is stereotypical, with loss of cells from blood. The study of the BAL allowed visualisation of an enrichment in the site of inflammation at the same time. That is an important finding as loss from blood could be linked to recruitment to tissues or activation-induced cell death (or both). The findings of enriched and activated MAIT cells in BAL suggests at least recruitment is occurring and places the cells at the site of inflammation.

These studies have revealed also consistent changes in activation status as judged by upregulation of a number of transcripts and surface molecules, amongst the most consistent of which is CD69. This was seen across all cohort studies including in patients with cancer [59] and pregnant women [55]. Normally levels of CD69 on MAIT cells in blood are low, although there is some background, which may represent recent activation or potentially recirculation of cells out of tissue. In the context of Covid-19 there is a consistent correlation between the level of CD69 expression on MAIT cells and disease severity in almost all studies. One interesting feature is that in patients who are ventilated on ICU with critical disease, where MAIT cells are depressed in the circulation and highly activated as described, there is still quite a range of CD69 expression. Exploring associations with clinical outcome, in one study a clear relationship was seen in that higher CD69 expression on MAIT cells was associated with fatal disease [58]. This was not so clearly shown with any of the other T cell subtypes seen (e.g. iNKT, Vδ2, bulk T cells), even though they showed some features of activation in this infection. It was not however unique to Covid-19 as similar features were associated with more severe outcomes in the influenza comparator group.

The implications of these studies of activation are two-fold: firstly, in theory at least activation of MAIT cells (e.g. via CD69 expression) could be used as a biomarker of disease with worse clinical profile (the impact was seen in a model where other clinical features were taken into account). The second implication is that activated MAIT cells could potentially contribute to disease. This could potentially be through secretion of relevant cytokines – such as GM-CSF which is known to be pathogenic in Covid-19 and is a drug target [63]. It could also be via killing of infected cells (or bystander cells), as suggested by one study [52]. Certainly, activated MAIT cells are “licensed” through upregulation of Granzyme B – which also serves as a marker of activation [64]. Analysis of killing of infected cells revealed cytolytic activity – driven by IL-18 but also unexpectedly impacted by MR1 blockade. The role of MR1, TCR and other surface triggers in virally induced MAIT cell activation is an area that clearly requires further exploration. Clearly, it is possible to activate MAIT cells in the absence of MR1 as shown in vitro and also in vivo [17] – but MAIT cell activation is typically a multi-component process, so this does not exclude an additional role for MR1 signals. The nature of the ligand and the overall mechanisms that lead to triggering of granule release (which is typically associated with TCR triggering) and also the pathways causing target cell death all need future study based on these data.

What are the mechanisms leading to such strong activation of MAIT cells in Covid-19, especially in the most severe illness? Many cytokines activate MAIT cells – typically in combinations. IL-18 has been seen as a strong driver of MAIT activation in almost every viral setting and Covid-19 appears to be no exception with some in vitro data to confirm this [52]. Other cytokines which could contribute include IL-15, IL-12 and TNF (reviewed in [12]. Defining which of these are really contributing to the activation/loss of MAIT cells in blood is quite complex – although gene expression studies can give some indication. In the critical care study described above there was a clear difference in the drivers of activation linked to fatal vs non-fatal outcomes as judged by analysis of scRNASeq data [58]. In those with a non-fatal outcome – and lower MAIT cell activation - there were features driven by IFNα. In contrast, in those with a fatal outcome and very high levels of MAIT activation there were multiple distinct pathways involved. This latter finding was not unique to MAIT cells – other cell types showed the same IFNα vs multi-cytokine patterns, which is not surprising considering they share the same environment. However, it was not seen in influenza, where survival was not clearly linked to an IFNα-dominant signal. Other transcriptional studies of MAIT cells in Covid-19 have also revealed distinct pathways driven in more severe disease, including cytotoxicity [57].

The key role of IFNα signalling in clinical outcomes in Covid-19 has been highlighted by studies of host genetics (e.g. GenOMICC [65]) and the link with anti-IFNα antibodies [66]. This transcriptional insight suggests that MAITs could be acting as biosensors of the IFNa-to-IL-18 switch observed and potentially amplifying these pro-inflammatory signals in the most severe cases [52]. It seems likely therefore that at this stage they are involved in rather than protect from pathology – although whether in the lung tissue (or other affected organs) this is as a critical intermediary, an innocent bystander or a major culprit still needs further examination in clinical and pre-clinical studies.

One set of questions that emerges from these overlapping studies is whether MAIT cells are behaving differently from other cells in Covid-19, and also how specific any changes are for this infection compared to others. Some potential answers come from unbiased multi-omic studies of Covid-19 and controls [53], [56], [59], including the COMBAT study, which examined sepsis and influenza controls in parallel [67]. This study – based on CyToF, FACS, scRNASeq, bulk RNASeq, proteomic, ELISA and ATAC-seq datasets - also showed the very strong association between MAIT decline/activation in blood and disease severity in both Covid-19 and sepsis and changes in MAIT populations came through as associations with severe disease in a hypothesis-free approach. However, there were not substantial differences between the Covid-19, sepsis and influenza groups. These data indicate that MAIT behaviour is distinctive in severe disease, with clearly evident changes even when all measurable cell populations are assayed – but that this occurred across a range of aetiologies. Further work needs performing to interrogate this and other datasets for the transcriptional drivers of this behaviour, but the analysis to date indicated upregulated signalling via TNF and KRAS [67].

The sharp focus on the immunology of Covid-19 has led to an explosion of data on MAIT cell dynamics, phenotypes and function in severe viral illness. This includes an insight into the role of sex, which is known to play an important part in clinical outcome [53]. Datasets generated from males vs females suggested a more dynamic MAIT cell response in females (who also have higher MAIT cell levels than males on average). The reasons why females have a better MAIT cell response have not been delineated but meanwhile the conclusion from this is that early MAIT cell responses in females might contribute to the better overall response to the virus – potentially paralleling the results from severe influenza already discussed.

If this is true, one might predict that baseline differences in MAIT cell levels (or functionality) could impact on the subsequent control of the virus. While datasets of pre-infection MAIT levels linked to outcome have not been published, one approach was to look in the recovery phase at correlates of severity, considering only populations which tended to return to the set-point [68]. Interestingly, a lower MAIT cell level was one such correlate, consistent with the idea that they play a protective role in acute disease. If this is the case it would also explain some of the increased disease risk associated with obesity [69] as well as age, where MAIT cell decline has been observed [70], [71].

Overall a number of consistent features linking MAIT cell status and clinical status are now emerging across Covid-19 studies and indeed across other viral infections. These are reviewed in Table 1.

**Table 1:** Clinical correlates of MAIT cell function and frequency.

Virus	Measurement	Clinical correlate	Reference (s)
DENV	MAIT function (IFNγ)	• sCD14 (microbial translocation, barrier function)	[48]
HBV/HDV	MAIT frequency (blood)	• sCD163 (macrophage activation)	[41]
HCV	MAIT frequency (liver)	• Liver fibrosis (ISHAK) • Liver inflammation (HAI)	[36]
HIV-1(acute)	MAIT activation	• sCD14 (microbial translocation, barrier function)	[47]
IAV(H7N9)	MAIT numbers	• Disease severity: time until recovery and survival	[49]
PUUV	MAIT activation	• Disease severity: inflammatory markers	[15]
SARS-CoV2	MAIT activation (CD69, PD-1 and CTLA-4)	• Disease severity: more severe disease and mortality	[51], [54], [72]

MAIT cell frequency, function and phenotype correlate with clinical parameters and disease outcome in different viral infections. Clinical parameters correlating positively with the indicated MAIT measurement are marked in blue, while such showing a negative correlation re marked in red.

## 5 MAIT cells and vaccines

Another, related, area where some new data has shed light on the role of cytokine-driven MAIT cell activation is in vaccines. The major vaccine platforms for Covid-19 have been those based on mRNA vectors and adenovirus vectors alone or in combination [73], [74], [75], [76]. The latter have been studied extensively for many years as vaccine candidates in infectious diseases and cancer [77]. However, many basic aspects are not understood, including the earliest events leading to priming. This is of interest because adenoviral vectors generate particularly strong priming of CD8 + T cells in humans, which is uncommon for virally vectored vaccines, and also because there are a wide variety of adenoviral subtypes which can be used for vaccines in their replication-incompetent forms, and these differ widely in their immunogenicity as well as their ability to trigger innate responses. A study of the role of MAIT cells in adenoviral vectors revealed that in humans and mice these are strongly activated by certain adenoviral vectors, but less so by others, especially those in Clade C (which includes HuAd5, a very commonly used vector). Activation was driven by IFNα and IL-18, but interestingly a 3rd signal was required [78]. This was derived from TNF, released from monocytes, in turn stimulated by IFNα released by plasmacytoid DCs. Blockade of TNF abrogated IFNγ release by MAIT cells in vitro and markedly altered the transcriptional pattern of activation of murine MAIT cells in vivo. The important feature of MAIT cells in this study was their role in CD8 + T cell priming – mice lacking MAIT cells showed reduced priming using vaccine vectors based on non Clade C viruses (e.g. ChAdOx1, as used in the Covid-19 vaccine) [78]. The mechanism for this is not yet defined, but likely includes a role for lymph node associated MAITs given this is the site of priming. MAIT cells are not enriched at this site but are readily detectable and strongly activated by the vaccines in vivo. Their transcriptional profile includes a range of chemokines which could play for example a role in CD8 + T cell or APC recruitment.

Much less is known about the role of MAIT cells in other vaccines. It is potentially the case that other virally vectored vaccines, such as poxviruses, activate MAIT cells as they will release overlapping cytokine blends – but the overall role of MAIT cells in priming would need examination in each case. Microbially based vaccines such as BCG could also function via a MAIT cell dependent pathway using both cytokine and TCR triggers, although in theory pre-existing MAIT populations could limit BCG replication and therefore restrict immunogenicity. Vaccine adjuvants used with inactivated or protein vaccines could also trigger MAIT cells via TLR pathways [79].

Vaccines based on mRNA have rapidly emerged as potent tools in the Covid-19 pandemic, but currently little is published on MAIT cell activation. These vectors have been available for some time[80] but more recently adapted so their RNA does not trigger inflammatory responses (largely IFNα) in the host, by chemical modification to avoid TLR7 recognition and careful removal of double-stranded RNA [81]. Given the very strong role of IFNα in driving responses in viral infections and adenoviral vectors, triggering of MAIT cells is unlikely initially, and has not been reported. However, in the boosting phase, where more cytokines are released, activation of a range of cell types has been reported, including NK cells and an NK-like T cell which phenocopies MAIT cells [82]. Their overall role in immunogenicity of mRNA vectors in not known although intriguingly a recent report indicated lower immunogenicity in recipients who had reduced MAIT cell frequencies and responsiveness [83]. Given that MAIT cells do vary widely between individuals and decline with age as already discussed, whether baseline MAIT cell frequency or functionality impacts on vaccine responsiveness (for any of the vaccines discussed above) is an interesting, important, and hopefully answerable question.

In the context of vaccination, it is also noteworthy to mention that MAIT cells, as well as other innate-like T cells, where shown to be capable to support antibody production and provide help to B cells in various settings. Studies in murine models of systemic lupus erythematosus (SLE) showed that the presence of MAIT cells is associated with higher levels of autoantibodies and that the impact of MAIT cells is dependent on CD40-CD40L interaction suggesting a role for MAIT cells in providing help to B cells in this setting [84]. Importantly, it was further shown that animal and human MAIT cells themselves are capable of producing several cytokines known to stimulate B cells including CXCL12, CXCL13, IL-6, IL-10 and IL-21 and are capable of directly supporting B cell differentiation *in vitro* and *in vivo* [85], [86], [87]. Recent data actually described a small, dedicated subset of MAIT cells with a phenotype (expression of CXCR5, BCL-6, ICOS, high levels of PD-1) resembling classic follicular helper T cells (TFH) in human tonsils [88]. This is in line with description of TFH-like subpopulations of iNKT and γδT cells that were shown to be capable of providing B cell help and drive antibody production [89], [90], [91], [92], [93], [94], [95] although further work is required to further define this rare subset in tissues.

An interesting question regarding MAIT cell help is what kind of antigens MAIT-induced antibodies would recognize. While older studies interestingly linked MAIT cells to polysaccharide-specific but not peptide-specific IgA responses [96], [97], Jensen et al. reported only a moderate impact of transferred MAIT cells on such antibodies in a *Vibrio cholerae* infection model [88] and to date, no further studies have looked into the matter.

Interestingly, it was shown that CXCR5 + γδT cells besides helping B cell directly, are also capable of supporting the differentiation of conventional CD4 T cells into TFH cells in mice [98]. Given that the functional capacities of unconventional T cells show a large degree of overlap, it is intriguing to speculate if MAIT derived cytokines might be able to do the same and hence could indirectly contribute to the generation of a large variety of antibodies with different specificities.

One final question that needs addressing is how a specific role for MAIT cells can be observed in a viral/vaccine context when the pathways for activation are common to many unconventional T cells and also NK cells. This is an important question and indeed it seems likely that in many settings a non-redundant role would be seen, given the overlapping and compensatory nature of these populations. Potential answers lie in the distribution and also the timing. In the context of a non-replicating vaccine, there is a very short burst of cytokines and it may be that MAIT cells are very readily triggered by this wave – in other words first cells off the blocks. Given the exponential nature of the priming process, very early events are likely quite dominant. Secondly, not all these cells are equally distributed. Clearly this differs between species so some caution is needed to move from mouse to man. If the primary signals lie in an environment relatively rich in MAIT cells compared to other potentially triggered subsets, then a much clearer role will be seen. These hypotheses are testable in future.

## 6 Open questions and future directions

**Fig. 1:**
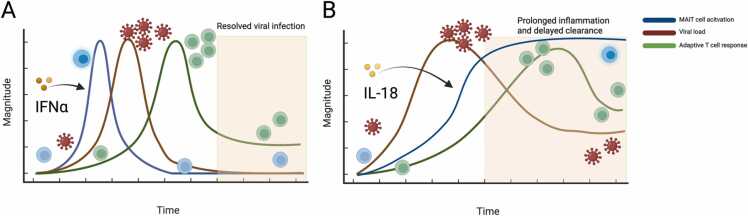
Differential infection kinetics impact on MAIT cells. In the context of acute infections (A), MAIT cells upregulate activation markers as the viral load increases but normalise in phenotype as soon as a robust adaptive immune response is mounted, and the viral infection is cleared. MAIT activation correlates with IFNα production and higher initial MAIT frequencies are correlated with fastened recovery overall implying a beneficial role of MAIT cells. In contrast, MAIT activation in protracted viral infections (B) is mainly driven by IL-18, appears to be long-lasting and is associated with more severe disease and worse clinical outcome. Figure was created with Biorender.com.

In this review we have discussed the most recent data on human MAIT cells in disease, which have been dominated by many studies of Covid-19. Some of these features could have been predicted from previous studies of viruses – but nevertheless they are very striking in different ways. The conclusions from these studies at one level are reassuringly very similar, and possibly only under the conditions of a pandemic would so many groups generate so many comparable datasets. Furthermore, the same conclusions regarding the behaviour of the cells have been reached using both targeted and hypothesis free approaches, including transcriptional studies. The conclusions appear to condense around a potentially early protective role for these cells in acute infection, and a potentially harmful role in protracted, more severe infection (Fig. 1). However, for the protection against Covid-19, as for influenza, there are many gaps in our knowledge – especially whether MAIT cells act directly or only via recruitment of other cell types, and what pathways really mediate protection e.g. in the lung. These are addressable given the availability of mouse models where MAIT cell behaviour in response to viruses. The same knowledge gaps also apply to their role in vaccine responsiveness, although likely in the lymphoid tissues rather than at epithelial sites. Given the enormous mass of data now suddenly available and the rising visibility of MAIT cells in these settings it is hoped that such gaps can be readily filled.
